# Are *In Vitro* Methods for the Detection of Endocrine Potentials in the Aquatic Environment Predictive for *In Vivo* Effects? Outcomes of the Projects SchussenAktiv and SchussenAktiv*plus* in the Lake Constance Area, Germany

**DOI:** 10.1371/journal.pone.0098307

**Published:** 2014-06-05

**Authors:** Anja Henneberg, Katrin Bender, Ludek Blaha, Sabrina Giebner, Bertram Kuch, Heinz-R. Köhler, Diana Maier, Jörg Oehlmann, Doreen Richter, Marco Scheurer, Ulrike Schulte-Oehlmann, Agnes Sieratowicz, Simone Ziebart, Rita Triebskorn

**Affiliations:** 1 Animal Physiological Ecology, University of Tübingen, Tübingen, Germany; 2 Faculty of Science, RECETOX, Masaryk University, Brno, Czech Republic; 3 Department Aquatic Ecotoxicology, University of Frankfurt am Main, Frankfurt am Main, Germany; 4 Institute for Sanitary Engineering, Water Quality and Solid Waste Management, University of Stuttgart, Stuttgart, Germany; 5 Water Technology Center Karlsruhe, Karlsruhe, Germany; Glasgow Caledonian University, United Kingdom

## Abstract

Many studies about endocrine pollution in the aquatic environment reveal changes in the reproduction system of biota. We analysed endocrine activities in two rivers in Southern Germany using three approaches: (1) chemical analyses, (2) *in* *vitro* bioassays, and (3) *in* *vivo* investigations in fish and snails. Chemical analyses were based on gas chromatography coupled with mass spectrometry. For *in* *vitro* analyses of endocrine potentials in water, sediment, and waste water samples, we used the E-screen assay (human breast cancer cells MCF-7) and reporter gene assays (human cell line HeLa-9903 and MDA-kb2). In addition, we performed reproduction tests with the freshwater mudsnail *Potamopyrgus antipodarum* to analyse water and sediment samples. We exposed juvenile brown trout (*Salmo trutta* f. *fario*) to water downstream of a wastewater outfall (Schussen River) or to water from a reference site (Argen River) to investigate the vitellogenin production. Furthermore, two feral fish species, chub (*Leuciscus cephalus*) and spirlin (*Alburnoides bipunctatus*), were caught in both rivers to determine their gonadal maturity and the gonadosomatic index. Chemical analyses provided only little information about endocrine active substances, whereas the *in* *vitro* assays revealed endocrine potentials in most of the samples. In addition to endocrine potentials, we also observed toxic potentials (E-screen/reproduction test) in waste water samples, which could interfere with and camouflage endocrine effects. The results of our *in* *vivo* tests were mostly in line with the results of the *in* *vitro* assays and revealed a consistent reproduction-disrupting (reproduction tests) and an occasional endocrine action (vitellogenin levels) in both investigated rivers, with more pronounced effects for the Schussen river (e.g. a lower gonadosomatic index). We were able to show that biological *in* *vitro* assays for endocrine potentials in natural stream water reasonably reflect reproduction and endocrine disruption observed in snails and field-exposed fish, respectively.

## Introduction

Endocrine disruptors (EDs) are substances which can affect the endocrine system by imitating or repressing body’s own hormones. Chemicals with endocrine potentials form a very diverse group and the number of chemicals known to cause endocrine effects in organisms is constantly increasing. This group includes for example synthetic estrogens, bioflavonoids, organochlorine pesticides, dioxins, furans, phenols, alkylphenols, polychlorinated biphenyls, phthalates, and brominated flame retardants. Also, naturally produced steroid hormones like 17β-estradiol (E2), estrone (E1), or testosterone, as well as phytohormones have the potential to affect endocrine systems in other organisms. However, natural endocrine-active chemicals are often less persistent than synthetic EDs [1].

Recently, a growing number of scientists, in particular toxicologists and ecologists, have pointed out the hazardous effects that different endocrine-active chemicals may have on the environment and animal and human health [2]. For example, many EDs are suspected to contribute to the development of breast cancer in women and prostate and testicular cancers in men, to reduce male fertility and to interact with the immune system [3], [4]. Disruptions of endocrine functions also occur in wildlife. Reduced fertility, abnormal development of embryos, feminization, and demasculinization are reported for birds, reptiles, mammals, and fish, while defeminization and masculinization are reported for gastropods (summarized in [5]). A number of distinct characteristics make EDs especially problematic. First, the wide range of effects caused by EDs makes it difficult to identify all hazardous effects. Second, low exposure levels are sufficient to cause serious consequences. For example, 17α-ethinylestradiol (EE2) is considered to be a very potent estrogen for fish; its lowest observed effect concentration for vitellogenesis in rainbow trout is 0.1 ng/L [6]. Therefore, already concentrations of estrogens and their mimics that are currently observed in freshwaters may impact the sustainability of wild fish populations [5], [7], even though direct evidence to relate endocrine disruption to wildlife population decline is rare [8], [9]. Third, many EDs are highly persistent, which often leads to long-term exposure. Once released into the environment, EDs may affect biota over many years, and it is difficult to assess these long-term effects with regards to the whole ecological community. Fourth, mixtures of EDs can interact, and thus either enhance or counteract the action of single substances. Studies on mixture toxicity offer increasing evidence that joint effects can occur when all mixture components are below levels at which individual chemicals cause observable effects [10], [11].

A main source for ED chemicals is the discharge of waste water treatment plants (WWTPs) into recipient waters. River pollution through waste water is especially relevant in areas with industry, high human population density, and/or intensive agriculture. Today, most waste water is treated in developed countries, but often endocrine disrupting chemicals cannot be completely removed by routine waste water treatment, and additional techniques to improve waste water purification are necessary [12]. Even in highly developed countries untreated waste water may be dumped into rivers when the capacity of WWTPs and stormwater overflow basins is exceeded during heavy rain events [13].

Given the evident relevance of EDs and the importance of WWTPs for their discharge into the environment, the present study assesses the effects of WWTPs on the water quality of two tributaries of Lake Constance, the Schussen and Argen rivers, as part of the “SchussenAktiv” and “SchussenAktiv*plus*” projects. As a first step, these projects examine the current ecological state in Schussen and Argen rivers. After different types and sizes of WWTPs at the Schussen are technically improved, these projects will then evaluate the effects of improved waste water treatment [14]. The present study reports the results on the water quality before the technical improvement of the examined WWTPs and consists of three main parts: chemical analyses of endocrine-active substances, a set of *in* *vitro* bioassays, and *in* *vivo* tests. These tests are employed to investigate estrogenic, anti-estrogenic, and anti-androgenic potentials and effects (and their temporal variability and trends) in the Schussen and Argen rivers and were jointly applied in view to elucidate the predictive value of chemical analyses or biological *in* *vitro* assays for organism-level endocrine effects in field-exposed biota.

Using chemical analyses, we focused on the identification of endocrine-active substances in surface waters and sediments. Previous chemical analyses detected up to 82 micropollutants, including EDs, in tributaries of Lake Constance. Thirty-five of these substances were found at ecotoxicologically relevant concentrations, for which effects on mortality, development, health, and reproduction of aquatic organisms cannot be excluded [15]. During the whole project we will analyse more than 150 micropollutants in waste water, surface water, sediments, and tissue samples [14].

Importantly, chemical analyses alone often provide very little information on the biological effects and do not take into account interactions among individual chemicals in mixtures. Therefore, we applied various bioassays to provide complementary information on biological potencies. Specifically, we use *in* *vitro* reporter gene assays detecting estrogen receptor (ER) or androgen receptor (AR) activation, and cell proliferation assays like the E-screen. These assays seem to be promising with respect to their mechanistic nature, relative simplicity, and potential high throughput [16]–[18]. Several field studies have demonstrated the diagnostic potential of bioassays, including studies with contaminated water and sediment samples [19]–[25].

However, sometimes results from *in* *vitro* assays are imprecise estimates for effects observed *in* *vivo* (see, e.g. [26]). For example, in a study on zebrafish [7], the relative estrogenic potency of EE2 that was observed was about 25 times more potent in *in* *vivo* than could be expected based on the *in* *vitro* results. Therefore, we complement our *in* *vitro* assays by using *in* *vivo* tests with mudsnails and fish. For investigations of native water and sediment samples in the laboratory assessing reproduction disrupting potentials, we used the freshwater mudsnail *Potamopyrgus antipodarum*, which has been shown to be a sensitive test organism responding to reproduction disrupting chemicals, including estrogens and their mimics. Such effects can be assessed by quantifying embryo numbers in the brood pouch [27]. As a second *in* *vivo* test for assessing endocrine effects, we evaluated expression of the egg yolk precursor protein vitellogenin (vtg) in juvenile brown trout. Normally, only female fish produce vitellogenin, which is estrogen-dependent. However, estrogenic xenobiotics can also act on the hepatic receptors to induce synthesis of vitellogenin in males and juveniles [28]. Therefore, vitellogenin levels in male and juvenile trout can be used as a biomarker of exposure to estrogen active substances in the environment [6], [28]–[32].

In addition, we examined feral fish (chub and spirlin) to determine their gonadal development and to assess if there are indications for endocrine disorders in the feral fish population.

**Figure 1:**
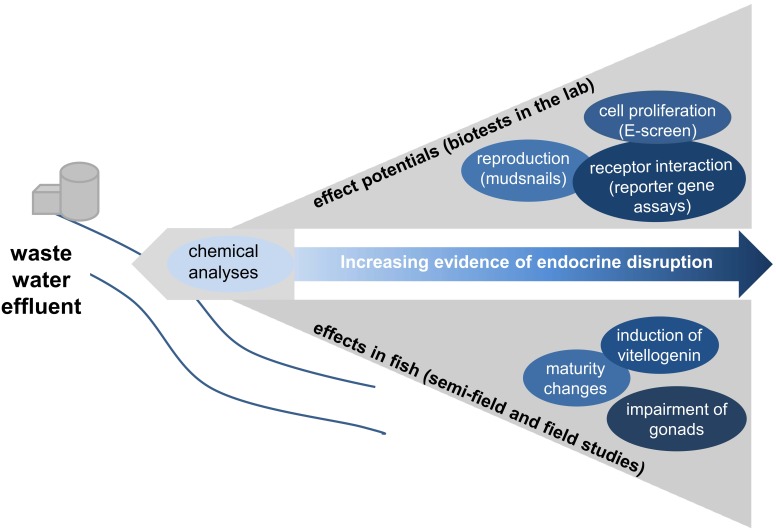
Model of the study design. This figure gives an overview of the study design and all performed analyses. Based on their results, we arranged the tests according to their evidence for endocrine disruption.

In contrast to large parts of extant literature, in this study we combined chemical analyses with *in* *vitro* assays and *in* *vivo* tests (Fig. 1). Thus, it was our aim to obtain a more precise and complete evaluation of endocrine activities at the Schussen and Argen rivers; in particular to investigate whether symptoms of endocrine disruption in field-living individuals are reflected by signals from *in* *vitro* laboratory assays or by the results derived from a detailed chemical monitoring programme.

## Materials and Methods

### 1 Study Sites, Bypass Systems and Exposure Experiments

**Figure 2:**
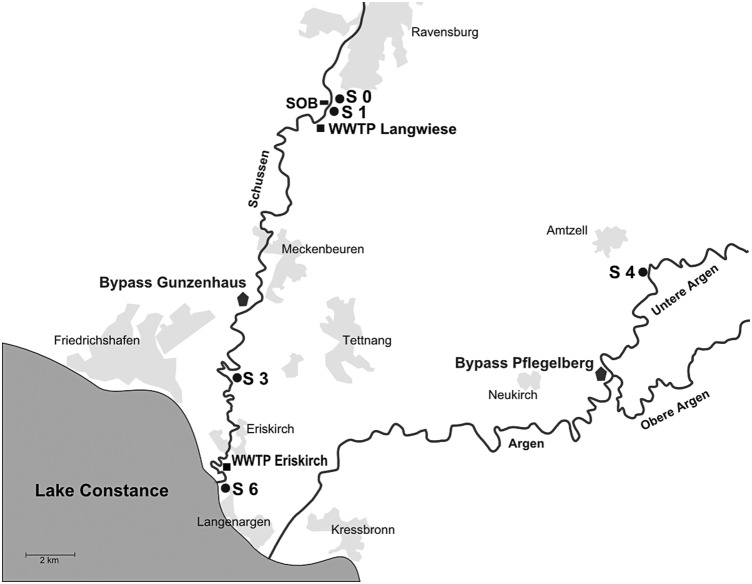
Location of the sampling sites and bypass systems at the Schussen and Argen rivers in Southwest Germany. Waste water treatment plant (WWTP) Langwiese and Eriskirch, as well as the storm water over-flow basin (SOB) at the Schussen. Geographic coordinates: S 0 = N47° 45′ 29.40″, E9° 35′ 21.78″, S 1 = N47° 45′ 19.22″, E9° 35′ 25.35″, S 3 = N47° 39′ 16.09″, E9° 31′ 53.35″, S 6 = N47° 37′ 4.73″, E9° 31′ 50.33″S 4 = N47° 44′ 20.46″, E9° 53′ 42.78″, bypass Gunzenhaus = N47° 40′ 44.00″, E9° 32′ 24.77″, and bypass Pflegelberg = N47° 39′ 11.21″, E9° 44′ 30.80″.

As a model region for a densely populated area, we investigated the Schussen river, a major tributary of Lake Constance. A total of 20 WWTPs and more than 100 stormwater overflow basins are connected to the Schussen [14]. Sampling site S 0 was upstream from one of the major waste water treatment plants (WWTP Langwiese) and a stormwater overflow basin, and site S 1 was located downstream from the stormwater overflow basin, but upstream from the WWTP Langwiese. Site S 3 was several kilometres downstream from the WWTP Langwiese, and S 6 was situated nearby the river mouth area at Lake Constance. Since a literature review by Triebskorn and Hetzenauer [15] showed less pollution at the Argen river, a reference sampling site, called S 4, was examined there. The location and sampling sites are shown in figure 2.

We collected water and sediment samples from all sampling sites. In addition, we analysed waste water (WW) from the WWTP Langwiese, which is one of the largest WWTP in the catchment area of the Schussen river (170,000 population equivalents). This WWTP has been upgraded with an active charcoal filter in autumn 2013. Table 1 shows all the sampling campaigns that we conducted from 2009 to 2013 (named from A to N).

**Table 1:** Dates of the sampling campaigns.

Code	A	B	C	D	E	F	G	H	J	K	L	M	N
Month	July	Oct.	June	Aug.	Oct.	May	July	Sept.	Oct.	May	July	Sept.	May
Year	2009		2010			2011				2012			2013

Two feral fish species, chub (*Leuciscus cephalus*) and spirlin (*Alburnoides bipunctatus*), were caught at sampling sites S 3 (Schussen) and S 4 (Argen) using electrofishing. In addition, we built bypass systems at both rivers, one downstream WWTP Langwiese at the Schussen and one at the Argen to simulate semi-field conditions (see figure 2 for the locations). These flow-through-systems were situated near the rivers, and river water was continuously passed through 250 L aquaria by a pump. At both bypass systems, we installed a sediment trap to guarantee similar concentrations of suspended particles. Technical supervision of water temperature, oxygen content, conductivity, and flow-through volume was carried out every 10 minutes, and failures were immediately reported by a short message. In these semi-field test systems, we performed exposure experiments with brown trout (*Salmo trutta* f. *fario*). The bypass systems allowed us to keep fish under controlled conditions that were close to their natural conditions (for a detailed description of the bypass systems, see [14]). As a negative control, we kept fish in 250 L aquaria under laboratory conditions in climate chambers at the University of Tübingen. Details for the exposure conditions of fish and catching procedure are described in 4.1. and 4.2.

#### Ethic statement

This study was carried out in strict accordance with German legislation (animal experiment permit nos. ZO 1/09 and ZP 1/12, field sampling permit AZ 35/9185.82–2, District Magistracy of the State of Baden-Württemberg).

### 2 Chemical Analysis of Endocrine-active Compounds

We analysed effluent samples from the WWTP Langwiese, surface water, and sediment samples from all sampling sites at different times (see Table 2). Immediately after extracting, 1 L of surface water sample and 0.2 L of WWTP effluent were preconcentrated by solid phase extraction (SPE) with a polymeric sorbent (Strata X, Phenomenex, Aschaffenburg, Germany) using an automated enrichment system (Autotrace, ThermoScientific). 4-n-nonylphenol and 17-α-methyltestosterone were added as surrogate standards prior the extraction process. We used 4-n-nonylphenol as a standard because literature did not describe its occurrence in aqueous environmental samples. The eluted samples were completely dried and derivatised by adding n-methyl-n-tri-methylsilyltrifluoracetamid (MSTFA) + trimethyliodosilane (TMJS) reagent. The analytical method is based on gas chromatography separation coupled to mass spectrometry detection (GC – MS, Agilent). Measurements were carried out in the laboratories of the Water Technology Center Karlsruhe (TZW, Karlsruhe, Germany). The procedures for sample preparation and analysis are based on DIN EN ISO 18857–1 (February 2007).

**Table 2:** Chemical analysis of water and sediment samples.

	2010	2011	2012
WWTP (Langwiese)	C, D, E	F, G, H, J	K, L
Site **S 0**	C		K, L, M
Site **S 1**	C		K, L, M
Site **S 3**	C	F	K, L, M
Site **S 4**	C	F	K, L, M

Sediment samples were also analysed by GC/MS. The sediment samples (1 g) were fortified with surrogate standards and extracted twice with 10 ml of acetone/cyclohexane (1∶10) in an ultrasonic bath for 15 minutes. Subsequently, the samples were centrifuged and the extracts were combined. The extracts were blown down to dryness and derivatised by adding MSTFA + TMJS reagent. Separation of the analytes was achieved by a Rxi - 5 Sil MS column (30 m x 0.25 mm, 0.25 µm) purchased from Restek (Fuldabrück, Germany). Transfer line temperature was 290°C. Temperature programme started with 120°C with holding time of 1 min was then ramped to 180°C with 15°C/min with no hold and then further ramped to 290°C with 5°C/min and 10 min hold. For the analysis a gas chromatograph 6890 coupled to a mass spectrometer 5973 (both Agilent Technologies, Waldbronn, Germany) were used.

### 3 Detection of Endocrine Potentials – *In vitro* and *In vivo*

#### 3.1 *In vitro* - E-screen assay

With the E-screen assay, we analysed effluent samples from the WWTP (Langwiese) and surface water samples from all sampling sites. The assay is based on the enhanced proliferation of human breast cancer cells (MCF-7) in the presence of estrogen active substances in the samples. The cell proliferation assay was developed by Soto et al. [17], optimized by Körner et al. [18], [33], and modified by Schultis (2005, unpublished data). To determine the estrogenic activity, the acidified (pH 2.5 – 3) water samples (1 L) were solid phase extracted (C18-cartridges, Varian Mega Bond Elut, 1 g). After drying the cartridges overnight by lyophilization and elution with methanol (2 x 5 mL), dimethylsulfoxid (DMSO, 50 µL) was added as a keeper to prevent loss of volatile substances. The MCF-7 cells were stored humidified (37°C, 5% CO2) in Dulbecco’s modified Eagle’s medium (DMEM) with fetal bovine serum and phenol red as buffer tracer (culture medium) and passed weekly. To accomplish the E-screen assay the cells were trypsinized and the culture medium was replaced by phenol red free DMEM with charcoal dextran treated fetal bovine serum (experimental medium). The cell suspension (75 µL, approx. 2300 cells/well) was plated into 96-well plates (Sarstedt, Newton, USA) and stored in the incubator for 24 h. For assaying the samples, dilution series were prepared (9 concentrations per sample) and added to the cells (8 wells per concentration). For providing a positive control (standard dose-response curve) the cells were exposed to a dilution series of 17β-estradiol (2.5·10–14 mol/L–2.5·10–10 mol/L). Neat experimental medium served as negative control (8 wells per plate). The E-screen assay was terminated after a five-day incubation time by removing the medium, washing the cells with phosphate buffered saline buffer and fixing them with trichloroacetic acid. After incubation (30 min; 4°C) the trichloroacetic acid was removed by washing the plates under a gentle stream of cold water. After drying the plates at 40°C the cell protein was stained with sulforhodamin B. After incubation (10 min) the dye was washed off with aqueous acetic acid (1%) and the plates were dried again at 40°C. The cell attaching dye was resuspended with tris-buffer and incubated (20 min; 4°C). The extinction was measured at 550 nm using a microtiter plate reader (MRX, Dynatech laboratories, Virginia, USA). Analysis of the dose-response curve was performed using the software Table Curve 2D (Jandel, San Rafael, CA).

The resulting estrogenic activity reflects a sum parameter over all estrogen active substances present in the samples and is expressed in concentration units of the reference substance E2 (17β-estradiol equivalent concentration, EEQ). The assessment of cytotoxicity in cells exposed to the investigated samples is important, because a high toxicity can overlay the estrogenic response. For example, if a water sample is both highly cytotoxic and estrogenic, the exposed cells should be triggered to proliferate but will not be able to do so because the cytotoxicity represses the cell proliferation. As a result, one will get an undersized “estrogenic response” from the test. Cytotoxicity was indirectly detected using different dilutions of the concentrated samples. The EC50 TOX value is the concentration of the examined sample in which 50% of the cells are able to grow. For illustration, we calculated the reciprocal values of the EC50 TOX values; high 1/EC50 TOX values represent a high cytotoxicity in the sample.

#### 3.2 *In vitro* - Cellular reporter gene assays for estrogens and androgens

With the reporter gene assays, we analysed effluent samples from the WWTP Langwiese and sediment samples from the sampling sites S 3 (Schussen) and S 4 (Argen). For effluents, one litre of each sample was filtered through a glass fiber filter using vacuum and extracted by SPE with SDB Waters Oasis (500 mg; columns were activated by 6 ml of methanol and equilibrated by 8 mL of distilled water, maximum backpressure was −30 kPa, and the flow rate did not exceed 10 mL/min). After SPE, the columns were dried, eluted with 6 mL methanol (no backpressure used), and concentrated by a nitrogen stream to final volumes which corresponded to 1200-times concentrated effluents. Sediment samples from the Schussen (S 3) and the Argen (S 4) were dried by freeze-drying (Christ lyophilization instrument), sieved through a 2 mm sieve, and 10 g were extracted for 1 h in 150 mL dichloromethane (automatic extractor Büchi System B-811). Extracts were concentrated by a nitrogen stream to the last drop and then dissolved in methanol. All extracts were stored at −80°C until testing.

To determine estrogenicity and antiestrogenicity, the human cell line HeLa-9903 was used according to the slightly modified protocol of US EPA [34]. Cells were grown in DMEM-F12 without phenol red (Sigma Aldrich, USA), containing 10% fetal calf serum, at 5% CO_2_ and 37°C. Once the cells reached about 80% confluence, they were trypsinized and seeded into a sterile 96-well plate at density 20 000 cells/well. For experiments, cells were grown in medium containing fetal calf serum treated with dextran-coated charcoal (which strongly reduces concentrations of natural steroids in the serum). After 24 h, the cells were exposed to the dilution series of the tested samples (6 different concentrations of each sample were tested), to the reference estrogen E2 (dilution series 1–500 pM E2) for the calibration, and to the blank and solvent controls (0.5% v/v methanol). To test for antiestrogenicity, the samples were co-exposed simultaneously with 33 pM E2, and the inhibitions of E2-induced responses were recorded. We used ICI 182,780 (7α,17β-[9-[(4,4,5,5,5-Pentafluoropentyl)sulfinyl]nonyl]estra-1,3,5(10)-triene-3,17-diol) as positive control. After the exposure, intensity of the luminescence was measured using Promega Steady Glo Kit (Promega, Mannheim, Germany). Effects on androgen receptor (AR) were evaluated with MDA-kb2 human breast cancer cell line [35]. Exposures were conducted in Leibowitz L-15 medium supplemented with 5% (v/v) stripped FCS at 37°C without added CO_2_. For testing antiandrogenicity, cells were seeded into 96-well plates (15,000 cells/well) in medium supplemented with 1 nM dehydrotestosterone (DHT) and exposed to a dilution series of extracts. After 24 h exposure, lysis buffer was added and luminescence measured after 30 min using 100 µL of substrate for luciferase according to Wilson et al. [35]. In all experiments, the solvent (methanol or DMSO) concentration did not exceed 0.5% v/v. Exposures were conducted for 24 h at 37°C.

#### 3.3 *In vivo* - Reproduction in potamopyrgus antipodarum

*Potamopyrgus antipodarum* (GRAY 1843), the mudsnail, originates from New Zealand. It can be found on soft sediments of standing or slowly flowing water bodies as well as in estuarine areas on the coasts at salinities up to 15‰ [36]. European populations consist almost entirely of female snails reproducing parthenogenetically. In Europe, male snails are found only very rarely [37], [38] and were never observed in our own laboratory culture. Although reproduction occurs throughout the year, the maximum offspring production occurs in spring and early summer, while the minimum is from autumn to early winter [39]. *P. antipodarum* performs a very distinct kind of brood care, termed ovovivipary [40]. The eggs develop in the anterior part of the oviduct, which is transformed into a brood pouch. After removing the shell of the snail, embryos can be accurately seen through the epithelia. By opening the brood pouch and subsequently removing the embryos and counting them, the reproduction success of each female is easy to determine.

Mudsnails for the testing of Schussen and Argen samples were taken from the laboratory culture of the Department Aquatic Ecotoxicology at Goethe University Frankfurt am Main, Germany. Tests were conducted according to the Standard Operating Procedure (SOP Part III: Reproduction test using sediment exposure) [41] and an OECD guideline proposal [42]. We measured mortality and the number of embryos in the brood pouch after 28 days of exposure.

Sediments from the two field sites S 3 and S 4, and from the effluent of WWTP Langwiese were analysed. Samples from the field sites, stored frozen (-23°C) until the start of testing, were obtained in seven independent sampling campaigns (C, D and E 2010, F, G, H and J 2011).

Samples were thawed at room temperature before testing and individual sediments were mixed with a stainless steel spatula. An aliquot of 100 g sediment (wet weight) was transferred into the test vessels (1 L screw-cap borosilicate glass). WW samples were thawed and 800 mL transferred into 1 L screw-cap borosilicate glass vessels. For the negative control (C) and the positive control (PC) an artificial sediment consisting of 95% quartz sand (grain size 50–200 µm) and 5% dried and fine-grounded beech leaves (*Fagus sylvatica*) was used per replicate. For the PC, the artificial sediment was spiked with a nominal concentration of 30 µg/kg of 17α-ethinylestradiol (EE2) in order to verify the estrogen-sensitivity of the test organisms. All sediment and WW samples were tested with two replicates, while four replicates were used for control groups (C and PC). All sediment samples, including C and PC, were covered with 800 mL of fully reconstituted water according to OECD [42]. Test vessels were aerated via a Pasteur pipette. Twenty adult snails with a shell height of 3.5 to 4.3 mm were used for each replicate vessel (static system, light-dark rhythm of 16∶8 h, 16±1°C, pH 8.0±0.5, oxygen content >8 mg/L, oxygen saturation >80% and conductivity 770±100 µS/cm). Only the WW samples were characterized by a slightly higher conductivity (797–1166 µS/cm). Water parameters were checked for each replicate at the beginning and end of the experiment and once a week during the experiment. Animals were fed three times a week with fine-grounded TetraPhyll® (0.2 mg dry weight per snail). After 28 days, all surviving snails were removed from the sediment and narcotized (2.5% magnesium chloride hexahydrate). The shell and aperture height were measured. The embryos were then removed from the pouch and counted, whereby shelled and unshelled embryos were distinguished.

### 4 Detection of Endocrine Effects – *In vivo*

#### 4.1 Vitellogenin detection in brown trout

Juvenile brown trout (*Salmo trutta f. fario*) were used as test animals for the active exposure experiments in 2011 and 2012. Freshly fertilized brown trout eggs were bought from a hatchery (2011: Störk, Bad Saulgau, Germany and 2012: Schindler, Alpirsbach, Germany) and exposure started 4 hours after fertilization in three different treatments (laboratory, bypass station at the Schussen and at the Argen). In each bypass station, 300 eggs were exposed in an aquarium with a constant flow-through rate of 12 l/min of water from the streams. As laboratory control, 300 eggs were held in an aquarium at 8°C in filtered tap water with a filter (Co.: JBL 1500e). A third of the water volume was exchanged once per week and, after the eying of the embryos, the light/dark photoperiod simulated field conditions. After hatching juvenile trout were fed by food for fry (Co.: BioMar, Biomar Inicio plus) and exposure continued till sampling (2011/12 exposure time: 99 days post fertilisation; 2012/13 exposure time: 111 days and 124 days post fertilisation). For vitellogenin analyses, larvae from each treatment were killed with an overdose MS-222 (tricaine mesylate, Sigma-Aldrich, St. Louis, USA), and the region between head and pectoral fin from each individual was placed in Eppendorf tubes, snap-frozen, and stored at −80°C.

All the following steps were undertaken on ice. Homogenates of juvenile trout were prepared by adding homogenization buffer (4-times the sample weight; PBS+2 TIU Aprotinin, C. Roth, Germany), mixing with a plastic pestle, centrifuging (10 min, 4°C, 20000×g (Eppendorf 5810R)) [31] and storing the supernatants at −80°C. As recommended by the provider of the test kit, a minimum of 1∶20 dilution was used. Each sample was tested in duplicate. In 2012/2013, the semi-quantitative ELISA test kit, which is recommended for vitellogenin analyses of salmonides, was used (Biosense Laboratories AS, Bergen, Norway; V01002402: Semi-quantitative vitellogenin Salmonid (Salmoniformes) biomarker ELISA kit). The enzyme activity (absorbance) which is measured in the assay is proportional to the concentration of vitellogenin in the sample (Automated Microplate Reader Elx 8006, Bio-Tek Instruments, INC., Winooski, Vermont, USA). Purified vitellogenin from Atlantic salmon (*Salmo salar*) was used as a positive control within every assay run as recommended by Biosense.

In 2011/12, we used a quantitative kit with a rainbow trout-specific antibody against vitellogenin (Biosense Laboratories AS, Bergen, Norway; V01004402: rainbow trout (Oncorhynchus mykiss) vitellogenin ELISA kit). As a pre-test to check the cross-reaction between rainbow trout antibody and brown trout vitellogenin, we analysed juvenile brown trout which we exposed for 16 days either to 40 ng/L EE2 or to clean water. Results of control fish showed 0 ng/L vitellogenin and EE2 exposed brown trout showed 2377±285 ng/L vitellogenin (each treatment: n = 6). This test showed that we are able to detect brown trout vitellogenin by using the rainbow trout specific antibody (rainbow trout kit).

#### 4.2 Maturity stage and gonadosomatic index (GSI) of feral fish

In the field, at sites S 3 (downstream from WWTP Langwiese, Schussen) and S 4 (Argen) two feral fish species, chub (*Leuciscus cephalus*) and spirlin (*Alburnoides bipunctatus*), were caught by electrofishing (for caught fish numbers see in the result section). Fish were killed with an overdose of MS-222 (tricaine mesylate, Sigma-Aldrich, St. Louis, USA), weighed, and measured lengthwise. The gonads were removed, weighed, and a small part of the middle part of the gonad was fixed in 2% glutaraldehyde in 0.1 M cacodylic acid for histological analyses. After embedding the fixed parts of the gonads in paraffin and cutting them in 3 µm slices, the slices were stained using two different methods (hematoxylin-eosin staining and alcianblue-PAS staining). Per fish 6 slices in three cell layers were evaluated by light microscopy and classified in 3 maturity stages according to Nagel et al. [43].

Female gonads:

Stage 1: Only oogonia or 90 to 100% previtellogenic or early perinucleolar oocytes present, <10% vitellogenic oocytes or yolk vesicle stadia
Stage 2: >10% vitellogenic oocytes or yolk vesicle stadia present, <50% mature oocytes with yolk and/or lipid
Stage 3: >50% mature oocytes with yolk and/or lipid present

Male gonads:

Stage 1: >80% spermatogonia, no spermatozoa present
Stage 2: <30% spermatozoa, residual spermatogonia, spermatocytes, and spermatids present.
Stage 3: >30% spermatozoa, residual spermatocytes, and spermatids present.

All statements refer to percentages of areas in the histological sections. The gonadosomatic index (GSI) was calculated according to Kang et al. [44]:
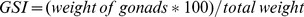



### 5 Statistical Analyses

#### 5.1 *In vitro* tests

The samples applied to the E-screen assay were quantified via the dose-response curve of the reference substance 17β-Estradiol (E2) and the curve of a dilution series of a sample extract. The estrogenic activity of the sample was calculated as the ratio of the EC50-values of 17β-estradiol (E2; positive control) and the dilution curve:
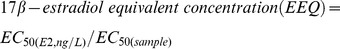



The limit of detection (LOQ) was defined as EC_10_ of the sample extract curve in comparison to the standard curve of E2. The LOQs depended on the individual concentration factor being used for the samples and were in the range of 0.01 ng/L–0.1 ng/L.

All samples analysed in the cellular reporter gene assays were tested in at least five different concentrations against each endpoint. Each treatment was performed in three replicates. The luminescence values measured in the estrogenicity and androgenicity assays were expressed as percentages of the maximum effect by subtracting the solvent control response and relating the values to the maximal response of standard ligand (E2_max_ for estrogenicity or DHT (dehydrotestosterone)_ max_ for androgencity). Maximum induction values as well as the shape of the curve differed among samples, thus equal efficacy or parallelism of the dose–response curves could not be assumed [45]. Final EEQ values (17-beta-estradiol equivalents) or DHT-equivalents were based on relating the amount of model ligand (E2 or DHT) causing 25% of the E2_max_ response (EC_25_) to the amount of sample causing the same response (determined from regression analysis). The EC values were calculated by nonlinear logarithmic regression of dose–response curve of calibration standard and samples in Graph Pad Prism (GraphPad Software, San Diego, USA). Assays enabled detecting estrogenic activity higher than 0.5 ng EEQ/L of effluent or 6 ng EEQ/kg of sediment. Antiestrogenicity and antiandrogenicity were expressed as the sample concentration that caused 25% inhibition of luminescence (IC_25_, g/ml) in the presence of competing ligand E2 (for antiestrogenicity) or DHT (antiandrogenicity). The IC values were determined on the basis of the linear regression models. The reciprocal value of IC_25_ is presented as 1/EC_25_ of the studied sample.

#### 5.2 *In Vivo* Tests

The statistical analysis of data of the reproduction test with *P. antipodarum* was performed using Prism®, version 4.03 software (GraphPad Software, San Diego, CA, USA). Normally distributed data (D’Agostino-Pearson test) with equal variances (Bartlett test) were tested with a one-way ANOVA with Dunnett’s post test for significant differences to the negative control (K). In all other cases, the nonparametric Kruskal-Wallis with Dunn’s post test was used. Mortalities, expressed as quantal data, were analysed using Fisher’s exact test.

Statistical analyses, which addressed the results of *in* *vivo* tests with fish, were performed with JMP 10.0 (SAS Systems, USA). Data were tested for normality using the Shapiro-Wilk W-test. If data were normally distributed the t-test was conducted, otherwise the Wilcoxon test or Steel-Dwass-test was used.

## Results and Discussion

### 1 Chemical Analysis

A total of more than 150 micropollutants, including endocrine-active chemicals, were analysed in more than 75 water and sediment samples. The following substances were always below their detection limits: 4-iso-nonylphenol, iso-nonylphenoldiethoxylat (detection limits: 25 ng/L) and all analysed polybrominated diphenyl ethers (BDE-100, −138, −153, −154, −183, −209, −28, −47, −66, −85, and −99; detection limits: 10 ng/L). Highly potent steroid hormones like 17α-ethinylestradiol and 17β-estradiol were not detected (detection limits: 1 ng/L). Our detection limits are high, and due to the fact that EE2 is biologically active in concentrations of 1 ng/L [46], biological effects of EE2 could be present although EE2 was not detected by our chemical analyses. In few samples, estrone was detectable but only in low concentrations up to 0.8 ng/L at S 3.

The phytohormone β-sitosterol was detectable in 5 out of 7 WW samples (max. 990 ng/L), in 1 out of 2 water samples of S 3 (360 ng/L) and in 2 out of 2 water samples of S 4 (max. 1.2 µg/L). 4-tert.-Octylphenol (in 3 out of 7) and bisphenol A (in 4 out of 7) were measurable in low concentrations in WW samples (detection limit: 5 ng/L). In the past, octylphenol occurred in surface water of the Schussen in concentrations up to 0,098 µg/L [15], which were close to the suggested target value of 0,1 µg/L for endocrine disrupting chemicals [47].

Sediment samples were analysed from campaigns C and F, and only low concentrations of β-sitosterol were found at all examined sampling sites. o,p-DDT, p,p-DDD, p,p-DDE and p,p-DDT were not detectable in any sediment samples (detection limit of 2 µg/kg dry weight). Analysed sediment samples of campaigns K, L and M showed a temporary occurrence of BDE-209 (max. 0.2 µg/kg) and di(n-butyl) phthalate (DBP) (max. 66 µg/kg) at sampling sites at the Schussen. Concentrations of perfluorooctanesulfonate (PFOS) and perfluorobutanoate (PFBA) were detectable only in few samples with concentrations up to 3.26 µg/kg.

In summary, the chemical analyses showed only few endocrine active substances in all investigated compartments. The phytohormone β-sitosterol was found in µg/L concentrations, but compared with synthetic or natural hormones, it is considered to be less potent by a factor 10^4^ [48]. This indicates that the risk of causing endocrine effects in animals living in the Schussen and Argen seems to be low. The fact that only few highly potent endocrine disrupting chemicals were found was unexpected (especially for waste water samples), because other studies (summarized in [15]) showed that there are detectable endocrine active substances, especially in the Schussen river.

### 2 Endocrine Effect Potentials

#### 2.1 E-screen assay

**Figure 3:**
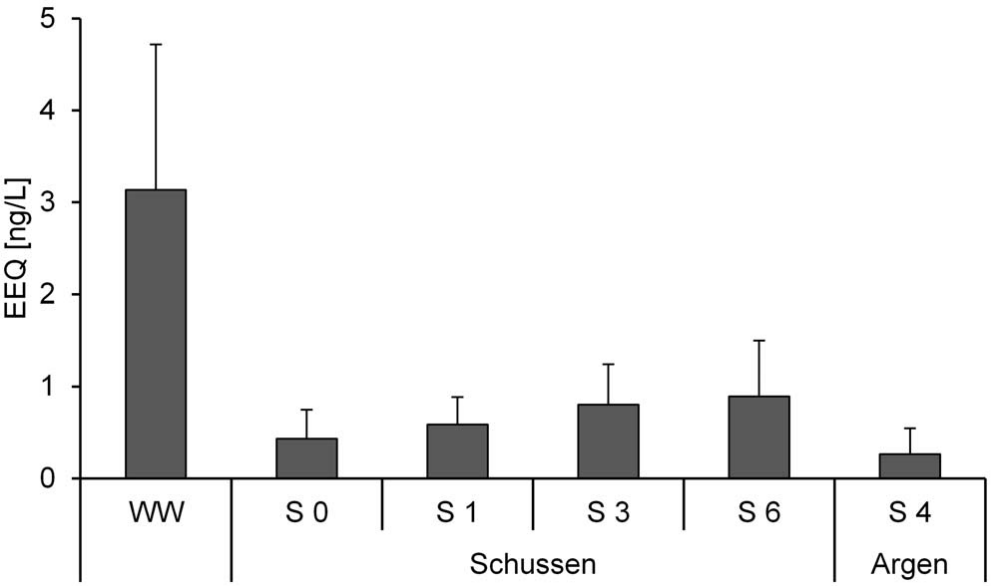
E-screen assay (estrogenic activity). Results of the E-screen assay expressed in 17β-estradiol equivalents (EEQ) in ng/L; means and standard deviation. Only data of samples which showed a low cytotoxicity (see figure 4) were used. WW (Waste water of WWTP Langwiese) n = 4, S 0 n = 5, S 1 n = 4, S 3 n = 7, S 6 n = 6 and S 4 n = 11).

**Figure 4:**
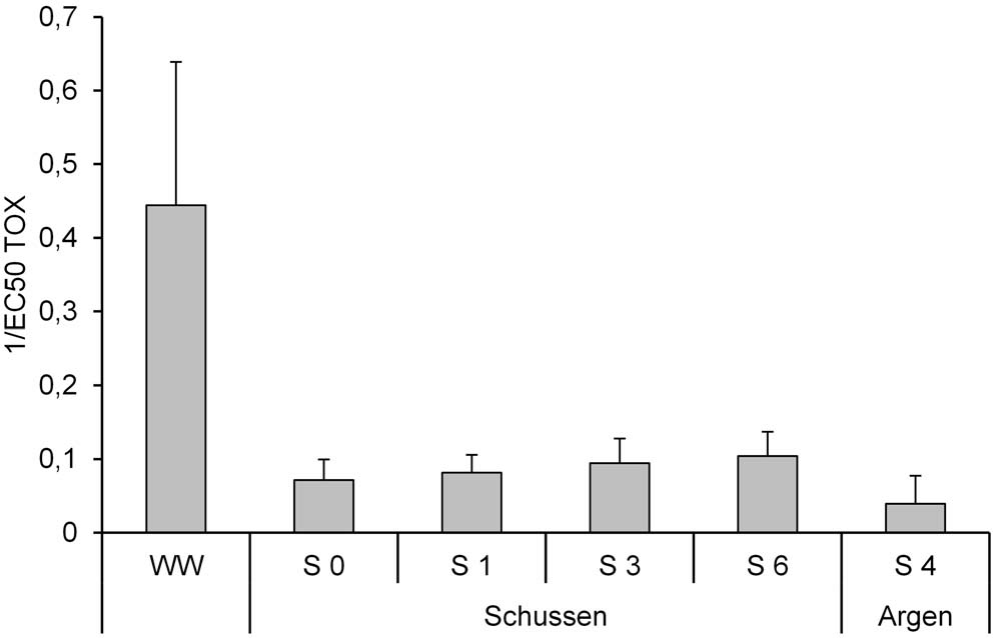
E-screen assay (cytotoxicity). Results of the E-screen assay regarding the cytotoxicity of the analysed samples. Expressed in 1/EC_50_ Tox (concentration in which 50% of the cells are able to grow) units; means and standard deviation. WW (Waste water of WWTP Langwiese) n = 9, S 0 n = 5, S 1 n = 4, S 3 n = 11, S 6 n = 11 and S 4 n = 11.

Figures 3 and 4 show means of EEQ and toxicity from all samples of the campaigns in 2010 (sampling C, D, E), 2011 (F, G, H, J), 2012 (K, L, M), and 2013 (N). The highest estrogenic activity was measured in the WW samples with a mean of 3.1 ng/L EEQ. At the sampling sites downstream from the WWTP (S 3 and S 6), EEQs of about 0.8 ng/L were detected. The lowest estrogenic activity was measured at the Argen (S 4) with 0.04 ng/L EEQ. Variability of the estrogenicity caused by seasonal or event-triggered effects assume to the average EEQs. Despite of these variations the results clearly showed a higher pollution of the river Schussen. The results of the cytotoxicity tests correlated with the results of the E-screen assay. Highest toxicities were observed in the WW samples and we had to exclude 5 of 9 samples in the E-screen because the high cytotoxic activity compromised the sensitivity of the E-screen assay. Similarly, samples of S 3 (5 out of 11) and S 6 (6 out of 11) showed high cytotoxicity and were also excluded. In contrast, samples of S 0, S 1, and S 4 had no evidence of cytotoxicity. Therefore, the estrogenic activity at Argen (S 4) and at two sampling sites at the Schussen (S 0 and S 1) could be assessed as low, whereas the WW clearly showed the highest observed estrogenic effects. The sampling sites downstream from the WWTP (S 3 and S 6) were charged less with estrogenic compounds compared to the WW. Due to an overlay of hormone action by cytotoxic effects, it is likely that the estrogenic potential in our samples from WW, S3 and S6 was actually higher than what our results suggest. Previous studies have found estrogenic activities in upper ranges as the one we measured with the E-screen assay: for WW samples (6–11 ng/L EEQ in [23], [49]) and for rivers (4 ng/L EEQ in [49]). The EEQ values determined by E-screen in Schussen samples are clearly indicative of expected significant field effects as it was recently proposed [50]. The mean value of 3.1 ng EEQ/L is above the E-screen-specific Estrogenic Limits (ELs) suggested (higher than 2 ng EEQ/L [50]).

#### 2.2 Reporter gene assays

Estrogenicity: In the effluent samples studied, no or only low estrogenicity was detected (one sample in campaign D with 0.88 ng/L of E2 equivalents, see Table 3). Nevertheless, the value determined with this reporter gene assay may indicate effects in vivo as it is within the range (or above) the Estrogenic Limits recently suggested. A number of research studies provide information on the estrogenicity of contaminated effluents and waters. These include a recent EU-wide study of 75 WWTP effluents [51], which has demonstrated that 27 of the analysed WW samples show estrogenic activity above the detection limit of 0.5 ng/L EEQ and that, in positive samples, estrogenicity varies from 0.53 to 17.9 ng/L EEQ.

**Table 3:** Summary results of mammalian cell reporter gene assays.

SEDIMENT SAMPLES							
	2010			2011			
	C	D	E	F	G	H	J
**Estrogenicity - ** ***[EEQ - pg E2 equivalent/g dw]***							
Site **S 3**	18,0	40,8	54,5	n.e.	n.e.	n.e.	49,7
Site **S 4**	14,08	6,13	n.e.	n.e.	n.e.	n.e.	n.e.
**Antiestrogenicity index [g/ml]^−1^**							
Site **S 3**	511	-	645	602	437	840	719
Site **S 4**	408	-	n.e.	412	210	485	198
**Antiandrogenicity index [g/ml]^−1^**							
Site **S 3**	19,9	n.e.	n.e.	8,2	6,6	25,1	51,0
Site **S 4**	4,4	n.e.	n.e.	8,5	8,4	19,5	13,3
**EFFLUENTS (WWTP, Langwiese)**							
	**2010**			**2011**			
	**C**	**D**	**E**	**F**	**G**	**H**	**J**
**Estrogenicity** [EEQ - ng/L]	n.e.	0,878	n.e.	n.e.	n.e.	n.e.	n.e.
**Antiestrogenicity index** [1/IC25]	n.e.	n.e.	n.e.	n.e.	n.e.	n.e.	0,4
**Antiandrogenicity** [1/IC25]	n.e.	n.e.	n.e.	n.e.	n.e.	n.e.	n.e.

n.e. = no effect up to the highest tested concentration, i.e. 0.5 g sediment dw/ml or equivalent of 12x concentrated water.

- = no samples analysed.

For sediment samples, the HeLa bioassay shows a low estrogenic potential, referring to absolute values. However, the trend between localities is clear - much weaker effects were apparent at S 4 (Argen; only 2 positive samples, maximum 14 pg/g EEQ) in comparison to S 3 (Schussen; maximum up to 55 pg/g EEQ), compare Table 3. Comparable estimates for sediment samples for other studies are relatively rare. For Czech sediments, median values measured using MVLN cells were around 100 pg/g EEQ (with maxima around 500 pg/g) [20] and 4.7–22 pg/g [52]. In various European sediments (ESP, DE, CZ) values about 75–669 pg/g EEQ [53], in rivers in France up to 200–6430 pg/g EEQ [54] and in four Italian rivers (7 sites) values between 15.600±7.300 pg/g EEQ [55] were reported. In comparison with the absolute values of these studies, our data are within the range or lower.

Antiestrogenicity: In effluent samples - similar to estrogenicity – we recorded weak antiestrogenic effects: only a single sample shows a measurable effect (campaign J - antiestrogenic index 0.4 [g/ml]^−1^). With respect to sediments, antiestrogenic effects were observed in several samples. Similar to estrogenicity, more pronounced effects were detected in the Schussen river (S 3; maxima up to 840 of the antiestrogenicity index [g/ml]^−1^) in comparison to the Argen river (S 4; maxima up to 485 [g/ml]^−1^). Antiestrogenicity showed seasonal dynamics with lower levels in spring and higher ones in autumn (Table 3). Previously, seasonal dynamics were reported in antiestrogenicity as well, with values in sediments ranging from 35–153 [g/ml]^−1^ during spring to 250–1000 [g/ml]^−1^ during autumn [20]. There are only few studies assessing antiestrogenicity in sediments: in Italian and Tunisian sediments no antiestrogenic effects were found, whereas in 3 rivers from an agricultural area in Nebraska (USA) a strong inhibition of E2-induced effects was reported [54], [56].

Antiandrogenicity: For **effluents**, none of the samples showed antiandrogenicity up to the highest equivalent concentration that was tested (i.e. 12-times concentrated). To our knowledge, only few studies investigated antiandrogenicity of surface waters or effluents, and the values reported previously were highly variable. Previous works reported 438 µg/L of antiandrogen flutamide equivalents (FluEq) for a river in Italy [57] and in Chinese surface water antiandrogenicity ranged from 20 to 935 µg/L FluEq [58]. Statistical modelling of the 30 WWTPs from UK waters predicted antiestrogenicity in FluEq values ranging 0–100 µg/L (with median and average of 10 and 20 µg/L, respectively) indicating that chemical cocktails of both estrogens and antiandrogens may contribute to the wild fish feminization [59].

In sediments (see Table 3), several samples always showed stronger anti-androgenic effects at S 3 at the Schussen compared to S 4 at the Argen. No anti-androgenic effects were observed during two campaigns (D and E). In general, higher effects were observed at S 3. Nevertheless, all values were lower in comparison to contaminated river sediments studied before [20]. Because the LOEC for fish is 63–651 µg/L FluEq as summarized by Runnalls et al. [60], we rarely expect antiandrogenic effects of the tested water in fish. Antiandrogenicity of sediment samples was also determined in previous studies, but the reported effects cannot be directly compared due to the use of different expressions/units: in sediments from the Czech Republic, antiandrogenicity was observed but not quantified [61], [62]; in Italian sediments a maximum inhibition of - 20% of dehydrotestosterone was reported [63], and in French sediments 1.1–32.5 µg/g flutamide equivalents were measured [54].

#### 2.3 Comparison of *in vitro* assays

Effluents of the WWTP Langwiese showed a higher estrogenic activity in the E-screen (four samples with mean 3.1 ng/L EEQ; Fig. 3) than in the reporter gene assay (estrogenicity detected only in one sample: 0.88 ng/L of EEQ; table 3). Therefore, the five day proliferation E-screen test seems to be more sensitive for the estrogenic assessment in comparison with the 24-h gene activation assays. Due to the high cytotoxicity observed in effluents, at S 3, and S 6 in the E-screen, we contend that the real estrogenic pollution is higher than 3.1 ng/L for effluents of the WWTP Langwiese (similarly for sampling sites S 3 and S 6). We used the reporter gene assay to analyse sediment samples, but not for surface water. Similar to the water sample results (measured with the E-screen), sediments from the Schussen (S 3; maximum 55 pg/g EEQ) showed higher estrogenic activities than those from the Argen (S 4; maximum 14 pg/g EEQ).

When comparing our results for sediment and water samples, it was obvious that the sediment samples showed a higher estrogenic activity than the water samples. Note that measurements of surface water (by E-screen) and sediment samples (by reporter gene assay) are not directly comparable due to different endpoints (growth vs gene transactivation) as well as origin of the cell lines used (MCF-7 vs HeLa-9903 [17], [34]). Previous work showed that the reporter gene assay with HGELN cells (which are derived from the HeLa cells used in the present study) may be less sensitive than the E-screen (with MCF-7 cells) when indicidual compounds are considered [64]. However, interpretation of tests with complex mixture samples (as performed in the present study with effluents, waters and sediments) may be more complicated depending on the actual composition of the studied samples. For example, simultaneous presence of both estrogens and antiestrogens may induce different responses (both estrogenic and antiestrogenic, depending on the concentration ranges and ratios). In the present study, high antiestrogenicity was detected in studied sediments being systematically higher at the S3 site in Schussen river. These results suggest that estrogenicity could be underestimated, and might be even higher than measured by the reporter gene assay. This is in line with results of Peck et al. [65], who have suggested that riverine sediments are a major sink and a potential source of persistent estrogenic contaminants. A study at the Upper Danube River in Southern Germany with *in* *vitro* assays also showed that endocrine disrupting potentials were elevated in selected sediments and confirmed an accumulation of endocrine active substances in sediments [66].

To summarize, our *in* *vitro* assays showed apparent endocrine disruptive potentials at the Schussen and Argen. These potentials varied over time, and were more pronounced at the Schussen. The presence of cytotoxic and antiestrogenic potentials implies that direct estrogenic potentials at the Schussen might be underestimated.

#### 2.4 Reproduction in potamopyrgus antipodarum

**Figure 5:**
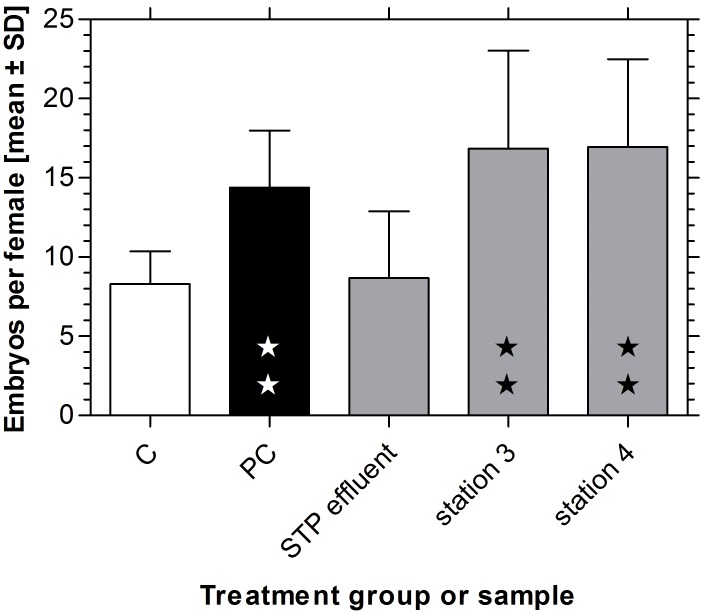
Reproduction test with the mudsnail. Means and standard deviation of the reproduction test with *Potamopyrgus antipodarum*. Total embryo number per female in negative (C) and positive controls (PC), in effluent water from the waste water treatment plant Langwiese (STP effluent) and in the two field sediments from sampling sites S 3 at the Schussen river and S 4 at the Argen river (station 3 and station 4) over the seven sampling campaigns. Asterisks indicate significant differences vs. C (one-way ANOVA with Dunnett´s multiple comparison test; p<0.01).

In order to assess the relevance of in vitro bioassays for the in vivo situation, we investigated reproduction in the mudsnail Potamopyrgus antipodarum. The overall mortality during the tests was quite low with a mean value of 5.8% and 9.5% for the negative and positive control, respectively. Although the mortality was nominally higher in the WWTP effluent samples (mean: 22.4%) and in sediments from the two field sites, S 3 and S 4 (15.2% and 13.7%, respectively), this increase was neither statistically significant when merging the values from all sampling campaigns nor for the single sampling campaigns (Fisher’s exact test, p>0.05). As the number of embryos in the brood pouch of *P. antipodarum* is positively correlated with shell height, all test animals were taken from a defined size class (3.5 to 4.3 mm shell height) at the start of the experiment. At the end of the experiment, differences in shell height between the treatment groups were very low (maximum difference of mean shell height: 4.01% between negative control and sediment from S 3 in August 2010) and not statistically significant (ANOVA, p>0.05). The average number of embryos in the brood pouch of females in the negative control group was 8.92, while females in the positive control group had a mean of 14.4 embryos in the brood pouch. This represents a highly significant increase of 74.5% (p<0.01, Fig. 5).

The mean embryo number of 8.67 in mudsnails that were exposed for four weeks to the WWTP effluent was not statistically significant different from the negative control. In contrast, the total number of embryos in female snails which have been exposed to the two field sediments from S 3 and S 4 was significantly higher than in the negative control with mean values of 16.9 and 17.0, respectively (Fig. 5). This increase by 104%–105% was even well above the level of the positive control (ANOVA with Dunnett’s post test, p<0.01). There was no significant difference in embryo numbers between females from the two field sediments.

It remains controversial as to whether reproduction in snails is regulated by an estrogen signalling pathway, homologous to vertebrates. Although there is broad empirical evidence that an exposure of caenogastropods and bivalves to estrogens and their mimics alters sexual differentiation and reproductive parameters, in some cases even at environmentally-relevant concentrations [27], [42], the observed effects on embryo numbers in *P. antipodarum* cannot univocally be attributed to estrogen signalling. This is because the endocrine systems of molluscs are insufficiently characterised and the precise mode(s) of action of endocrine active chemicals, including estrogens and their mimics are not fully understood. However, the significant increase of embryo production observed in the field sediments S 3 and S 4 is a clear indication for reproductive disruption with obvious potential for population level consequences [27], [67], [68].

The apical effects of an exposure to endocrine active chemicals in *P. antipodarum* have been reviewed by Duft et al. [27]. Exposure to various xeno-estrogens (BPA, octylphenol, nonylphenol, EE2) resulted in increased embryo numbers in the brood pouch of mudsnails. In the case of BPA, a stimulation of the reproductive output was noted in a sediment test with an EC_50_ of 5.67 µg/kg and an EC_10_ of 0.19 µg/kg after four weeks [69]. Exposure to BPA and EE2 via water was investigated by Jobling et al. [70], again resulting in a stimulated embryo production, with significant effects at a concentration of 5 µg BPA/L (NOEC 1 µg BPA/L) and 25 ng EE2/L (NOEC 5 ng EE2/L), respectively. A reproduction-disrupting effect of EE2 in *P. antipodarum* was confirmed by Sieratowicz et al. with a LOEC of 50 ng/L and a NOEC of 25 ng/L [39]. Most of the observed concentration-response relationships for both compounds, however, were biphasic, with an inverted U-shaped curve [39], [70]. This is important for the interpretation of results from tests with reproduction disrupting chemicals or environmental samples with *P. antipodarum* because at very high concentrations, the stimulation of reproductive performance declines, and may even fall back to the level of the negative control. Corresponding observations have been made in several other studies with snails [67], [69], [71]–[73]. They can be explained by a dominant stimulating effect of these reproductive disrupting test compounds at low concentrations and a decrease in embryo production due to their general toxicity at higher concentrations.

Therefore, the significantly enhanced embryo numbers in mudsnails exposed to the field sediments from S 3 and S 4 indicate the presence of reproductive disrupting compounds. The effects at both rivers are higher than the effects in the positive control with a concentration of 30 µg EE2/kg, which indicates severe pollution by reproductive-disrupting compounds in the sediments of both rivers. In contrast, the lack of significant differences in embryo numbers between the WWTP effluent and the negative control is not necessarily evidence for a lack of such compounds in the waste water. In complex environmental samples, the presence of reproduction-toxic substances may compensate for the effects of estrogens and other disruptive compounds on embryo production in a way that stimulating effects can be completely masked. It is also possible that, at high concentrations of reproductive-disrupting compounds in waste water, the number of embryos is again reduced to the negative control level due to the already discussed biphasic curve of the concentration-effect relationship.

Galluba & Oehlmann [24] applied the *in* *vivo* reproduction test with *P. antipodarum* and the yeast estrogen screen (YES) as an *in* *vitro* assay in parallel for 50 sediments from smaller rivers and creeks. It was shown that 54% of the sediments exhibited a promoting effect on snail reproduction and also showed an estrogenic activity in the YES while 82% of the samples which were active in the YES caused an increased snail reproduction. Despite this coincidence, the Spearman correlation between EEQs and embryo number in the snails was not significant because sediments with the highest EEQs in the YES caused no or little increase of embryo numbers. The lack of a significant correlation between the two systems may reflect the difference by which estrogens are acting in the yeast cells compared to how they are acting in the snail. Alternatively, it may be an indication that embryo numbers had returned to control levels at very high exposure to reproductive-disrupting compounds, reflecting the biphasic concentration response of the snails.

Galluba & Oehlmann [24] also discussed the possibility that lower embryo numbers in the artificial control sediment may reflect sub-optimal conditions for the development and reproduction of the snails. However, if embryo numbers in the tested field sediments are not compared to the artificial control sediment but to a natural reference sediment with no measurable estrogenic activity in the YES, an identical number of sediments turned out to exhibit significantly more embryos. This shows that reproduction in *P. antipodarum* is almost identical in natural sediments without estrogenic activity and in artificial sediments so that alternative explanations for enhanced embryo numbers such as the supply of more or better suited food can be ruled out.

Previous studies have pointed out that an increase in reproductive output in snails can have an adverse effect on the population [67], [69], [72]. A stimulation of reproductive output outside the main reproduction period may result in oviduct malformations as shown by Oehlmann et al. for *Marisa cornuarietis* [73]. Furthermore, the stimulation of reproduction outside of the breeding season is a waste of an organism’s energy reserves because offspring face less favourable environmental conditions for survival and growth during these periods [68]. Further possible consequences are a reduced somatic growth of adults and a decreased reproductive performance during the actual breeding season [71].

### 3 Endocrine Effects in Fish

#### 3.1 Vitellogenin detection in brown trout

**Figure 6:**
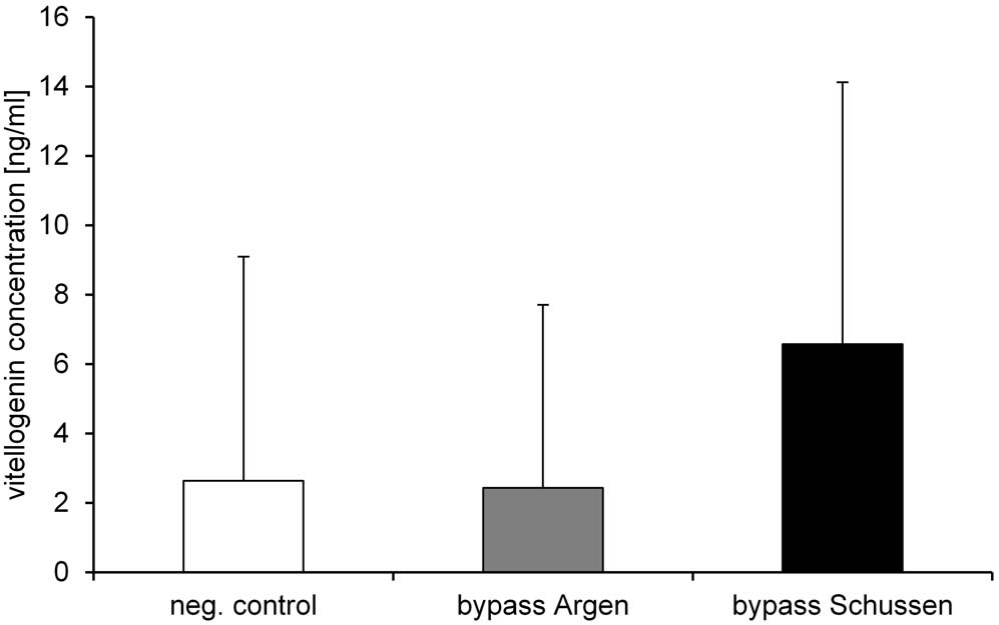
Vitellogenin in juvenile brown trout. Vitellogenin levels in homogenates of juvenile brown trout 99 days post fertilization in 2011/2012, means and standard deviation. Analysed by Biosense rainbow trout vitellogenin ELISA kit. Samples: Neg. control n = 6 (1 out of 6 pos. result, bypass Argen n = 10 (2 out of 10 showed a pos. result), bypass Schussen n = 10 (5 out of 10 showed a pos. result). No significant differences (Steel-Dwass-test: neg. control- bypass Argen p = 1,00, neg. control- bypass Schussen p = 0,5787 and, bypass Schussen- bypass Argen p = 0,4030).

In 2011/2012, juvenile brown trout, which were exposed at the bypass stations for 99 days after fertilization, showed higher average vitellogenin levels at the Schussen bypass compared to the Argen bypass and the negative control (Fig. 6). However, the differences were not significant. We analysed the samples with a kit that is specific for rainbow trout. Auxiliary tests indicate that the antibody cross-reacts more weakly with brown trout vitellogenin. Therefore we exposed juvenile rainbow and brown trout for 16 days to 40 ng EE2/L. After the exposure, we measured an average vitellogenin level of 2377 ng/L in the brown trout but found a higher average vitellogenin level of 279988 ng/L in the rainbow trout (while we analysed six brown trout samples, we were only able to analyse two rainbow trout samples because the others showed a strong reaction that exceeded the allowed extinction level of the assay). Given the difference in the ways the antibody binds with vitellogenin in brown and rainbow trout, we conjecture that the actual vitellogenin levels in juvenile brown trout were higher than shown in figure 6. Estrogen active compounds in the Schussen are likely causes for the increased vitellogenin levels. Vitellogenin levels in trout exposed at the Argen were lower compared to those from the Schussen, but not significantly so (p = 0,4030). This might result from the lower anthropogenic pollution of the Argen river [15]. Trout exposed at the Argen showed vitellogenin levels comparable to those of the negative control (p = 1,00).

**Figure 7:**
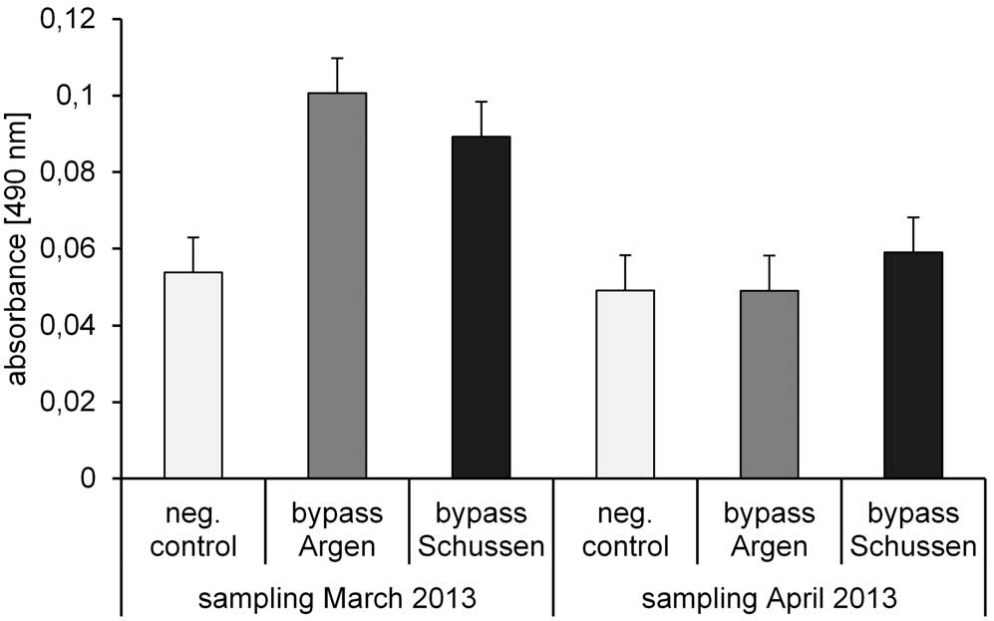
Semi-quantitative vitellogenin detection in juvenile brown trout. Absorbance measured in homogenates of juvenile brown trout 111 days post fertilization and 124 days after fertilization exposed in 2012/2013; means and SD. Each sampling analysed with one semi-quantiative vitellogenin salmonid (Salmoniformes) biomarker ELISA kit (enzyme activity = colour intensity is proportional to the concentration of vitellogenin in the sample). Samples March 2013: Neg. control n = 5, bypass Schussen n = 7, bypass Argen n = 6. Significant differences with Steel-Dwass-test: neg. control- bypass Schussen p = 0,0159 and neg. control- bypass Argen p = 0,0221; * = p<0.05. Samples April 2013: Neg. control n = 12, bypass Schussen n = 12, bypass Argen n = 12. No significant differences with Steel-Dwass-test.

In 2012/2013, vitellogenin analyses in 111 day-old juvenile brown trout showed no significant differences between trout exposed at the Schussen bypass and at the Argen bypass (Fig. 7, sampling March 2013). However, the values recorded for the negative control were significantly lower than those of trout exposed at the bypass stations. For the analyses, we used the semi-quantitative ELISA optimized for salmonids. The cross-reaction of the monoclonal antibody, BN-5, with brown trout vitellogenin is strong and recommended for vitellogenin analyses with brown trout [74]. Given that the negative control showed significantly lower levels (Steel-Dwass-test: neg. control- bypass Schussen p = 0,0159 and neg. control- bypass Argen p = 0,0221), the vitellogenin production in our juvenile brown trout is likely caused by estrogen-like substances occurring in the Schussen and Argen. However, analyses of vitellogenin in juvenile brown trout from a second sampling (124 days of exposure; see figure 7, sampling April 2013) did not show any significant differences between all three treatments, and the vitellogenin levels were all in the range of the negative control.

A previous study conducted by Stalter et al. [31], showed a significant increase in the vitellogenin concentration (nearly 70 ng/mL compared to less than 10 ng/mL in the control) in yolk-sac rainbow trout which were directly exposed to WWTP effluents for 60 days. Other studies that examined WWTP effluents using sexually immature or male trout also showed a correlation between vitellogenin levels and WWTP effluents [6], [75], [76]. Another reason for the increased vitellogenin levels could be an immune response caused by pathogens occurring in the river water [77]. However, Zhang et. al [77] argued that juvenile fish are probably not able to produce vitellogenin as an immune response. Hence, we conjecture that mainly estrogens are responsible for the increased vitellogenin levels.

Overall, the vitellogenin levels we have detected were rather low compared to previous studies. However, these studies either exposed trout directly to WWTP effluents [6], [32], [78] or examined older feral trout [76], [79], [80]. We interpret our results as showing that an estrogenic pollution might be present in both rivers, but that concentrations apparently have varied and were able to induce vitellogenin production only in some cases.

#### 3.2 Gonadal maturity and gonadosomatic index of feral fish

**Figure 8:**
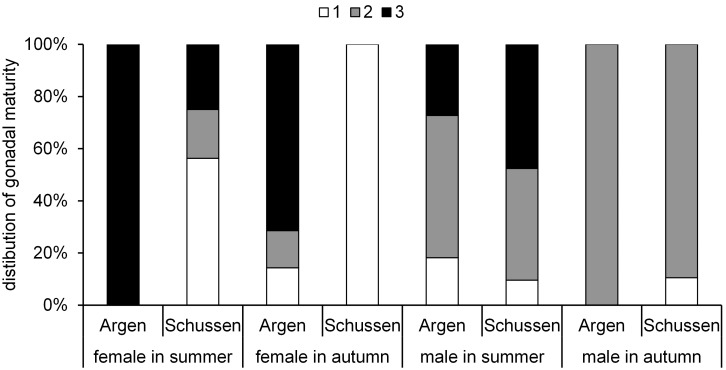
Maturity of chub. Distribution of gonadal maturity (stage 1 = immature; stage 2 = intermediate and, stage 3 = mature) of feral chub. 2009–2011. Females: summer Argen n = 2, summer Schussen n = 16, autumn Argen n = 7, autumn Schussen n = 12. Males: summer Argen n = 11, summer Schussen n = 21, autumn Argen n = 10, autumn Schussen n = 19.

Generally, the gonadal maturity levels (Fig. 8) we observed in chub were higher in summer than in autumn, which is due to the spawning season (April to June). After the spawning season, the gonadal maturity normally decreases until females generate new eggs and males build new spermatozoa. Female chub caught at the Argen showed an increased gonadal maturity compared to chub from the Schussen (Fig. 8), potentially reflecting a higher estrogenicity in the Schussen river or anti-estrogenic effects at the Argen river. We did not observe any differences in the gonads between male chub caught at the Schussen and Argen.

**Figure 9:**
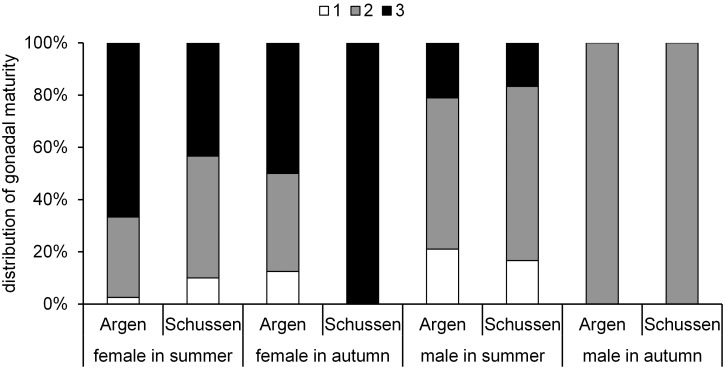
Maturity of spirlin. Distribution of gonadal maturity (stage 1 = immature; stage 2 = intermediate and, stage 3 = mature) of feral spirlin. 2009–2011. Females: summer Argen n = 35, summer Schussen n = 30, autumn Argen n = 16, autumn Schussen n = 7. Males: summer Argen n = 19, summer Schussen n = 3, autumn Argen n = 19, autumn Schussen n = 8.

In female spirlin from the Schussen and Argen rivers, differences in the maturity of gonads were low in summer (Fig. 9). In autumn, female spirlin caught at the Schussen showed a higher gonadal maturity than those from the Argen. Similar to the results obtained for male chub, we did not observe any differences in the maturity of male gonads between Schussen and Argen spirlin (Fig. 9). Because the spawning season for spirlin and chub is from April to July, it was expected that in autumn no spermatozoa would be detectable in the gonads of males and the maturity would be lower [81]–[83]. We did not find evidence for endocrine effects on male maturity in both rivers. Contrary to our results, a study on wild roach living in rivers receiving high amounts of effluents showed a progression of spermatogenesis mainly in males, whereas the females appeared to be less affected [84].

Female chub and spirlin reacted contrary to one another at the Schussen, whereas no difference between the two species could be observed at the Argen. At the Schussen, female chub (Fig. 8) showed a lower gonadal maturity but female spirlin (Fig. 9) a higher gonadal maturity compared to their respective conspecifics from the Argen. One possible reason for the observed differences is that the two species react differently to substances occurring in the Schussen. Although the water temperature at the Schussen is slightly higher than at the Argen in general, this is not a likely explanation for the observed differences. Higher temperatures could lead to faster gonadal growth and higher gonadal maturity [85], [86], and hence, cause a higher gonadal maturity of fish at the Schussen. However, as a higher maturity was only observed for female spirlin, the temperature is less likely to be the main cause for the observed effect.

**Figure 10:**
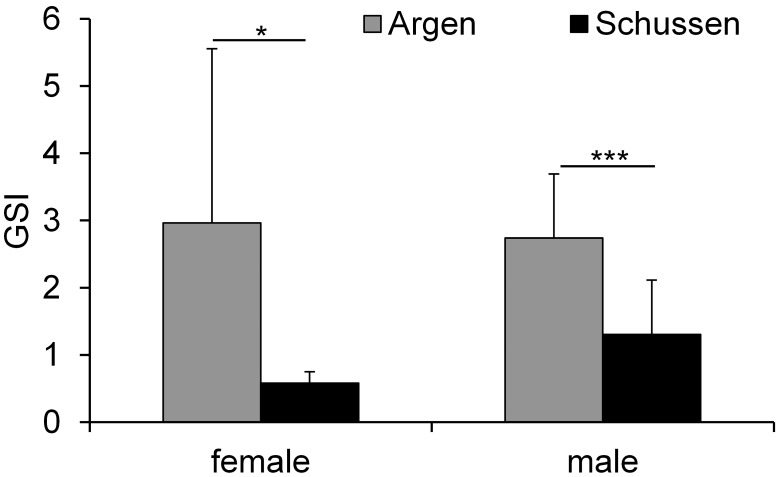
Gonadosomatic Index (GSI). Gonadosomatic Index of female and male chub caught in autumn 2010–2012 (sampling campaign E, J, and M); means and SD. Females: Argen n = 5 and Schussen n = 10. Males: Argen n = 12 and Schussen n = 16. Asterisks indicate significant differences between Schussen and Argen (* = p<0.05 and *** = p<0.001).

In spirlin we only determined the gonadal maturity because in the field it was technically not possible to weight small gonads exactly. In summer, we did not observe any differences in the GSI values for chub between the Argen and Schussen (results not shown). In autumn, female and male chub caught at the Argen showed a significantly higher GSI than chub from the Schussen (Fig. 10). Also, female chub from the Schussen showed a distinctly lower GSI than the lowest value reported for chub by Mert et al. [87]. This could be the result of substances and stress factors in the Schussen which hinder the development of the gonads and cause a delayed maturity. The fact that both sexes show a reduced GSI could be explained either by the simultaneous presence of anti-estrogenic, androgenic, and estrogenic substances or by a general worse health status of fish at the Schussen compared to fish at the Argen.

This is in line with several studies, which showed a reduced gonad growth in fish caught at polluted areas [88]–[90]. Investigations in brown trout also showed lower GSI values and vitellogenin production for trout caught downstream of WWTPs compared to trout caught upstream of WWTPs [91], [92]. A study about the interaction between 17β-trenbolone (TB) and EE2 in relevant environmental concentrations observed a decrease of the GSI of male eelpout after 21 days of exposure to EE2 alone or in combination with TB compared to controls [93].

### 4 Comparisons

Our *in* *vivo* tests revealed endocrine potentials/effects at the Schussen as well as at the Argen. The reproduction tests with *P. antipodarum* showed an equal increase in the number of embryos at both rivers, which were even higher than in the positive control (with a concentration of 30 µg EE2/kg). The vitellogenin levels we observed in juvenile brown trout also were increased at both rivers. Data of Jobling et al. [70] indicate that both, the nature of the response and the relative sensitivities to environmental estrogens, are comparable for *P. antipodarum* and rainbow trout. In concordance with this observation, our results for mudsnails were qualitatively in line with those for brown trout. We performed the tests with *P. antipodarum* with sediments only for 4 weeks, whereas the trout were exposed directly after their fertilization to the river water for several months. The results were stronger for mudsnails, despite the fact that exposure time were much longer for trout. A potential explanation for this is that sediments (used for mudsnails) showed high estrogenic and antiandrogenic activities (as indicated by the reporter gen assay), whereas in the surface water, which we used for the trout tests, only low estrogenic activities were detected (as revealed in the E-screen). While *in* *vitro* and *in* *vivo* (mudsnails and vitellogenin production) tests provided qualitatively comparable perceptions of the endocrine-disruptive activity, the results of the chemical analyses did not reveal the presence of endocrine substances at effect concentrations, probably because not even the broad range of substances analysed in this study could represent the plethora of potentially endocrine-active compounds which are supposedly present in the environment. Moreover, mixture effects might be important: even if individual compounds were not detected, a combination of substances at lower-than-detectable levels could cause an effect. The gonadal maturity examinations in feral chub and spirlin did not provide clear indications for the presence of endocrine active substances. Nonetheless, chub of both sexes caught at the Schussen showed reduced GSI values compared to those caught at the Argen. A mechanistic interaction of endocrine-active (androgenic and/or estrogenic) and toxic compounds, as indicated by the *in* *vitro* assays, could explain the reduced GSI values at the Schussen river.

When analysing effluents of the WWTP Langwiese, all our tests revealed temporary endocrine activities. However, chemical analyses revealed only low concentrations of chemicals like estrone, β-sitosterol, octylphenol, and bisphenol A, which fluctuated over time. We conclude that constant presence, but concentrations below the limit of detection, possibly, a variety of compounds were the reason why our chemical analyses did not succeed in detecting high numbers of potent endocrine disrupting substances. In addition, chemical analyses only reflect snap-shots of pollution (single sample from the field or 24 h sample of the WWTP effluent) whereas fish were exposed for several weeks (trout) or for their lives (chub, spirlin). Our *in* *vitro* assays indicated that the aggregate estrogenic potential was relatively low (0.9 to 3 ng/L EEQ), but high cytotoxicity (as indicated by the E-screen) and the existence of antiestrogenic potentials (as indicated by reporter gene assays) could probably lead to an underestimation of estrogenic potentials. Notably, mudsnails exposed to effluents showed no increase in the number of embryos compared to the negative control, but it is likely that estrogenic activities were masked by toxic substances, as indicated by increased mortality rates of mudsnails exposed to waste water, however, they were not significant higher. Our results suggest that the waste water has both estrogenic and toxic potentials.

## Conclusion

Using a biological and chemical monitoring programme at two German rivers, we investigated whether symptoms of endocrine disruption in feral animals are reflected by results obtained in biological *in* *vitro* assays and by chemical analyses. In our case, chemical analyses provided only little information about the occurrence of endocrine active substances. In contrast, the results of our *in* *vitro* assays showed endocrine-disruptive activities for most of the analysed samples, indicating that the discharge of treated waste water results in elevated endocrine-disruptive potentials. Similar results were obtained *in* *vivo* using mudsnail reproduction tests and measuring GSI values of feral fish. In contrast, vitellogenin levels of trout and the maturity of feral fish showed only a slight indication of estrogenic activities.

Our multiple testing approach revealed that the E-screen assay reports higher estrogenic activities compared to the reporter gene assay (for waste water samples), which suggests that the E-screen assay was more sensitive in our analyses. Furthermore, it showed that *in* *vivo* tests with mudsnails alone would have led to an underestimation of the estrogenic activity of the waste water samples.

Our results imply that an interpretation of individual test results can be questionable, because different conclusions could be drawn from the results (e.g., as toxic effects might overlay endocrine effects), and an over- or underestimation of the endocrine pollution might result. We therefore propose a combination of *in* *vitro* and *in* *vivo* tests supported by advanced targeted instrumental analyses to assess endocrine pollution in rivers. The individual test results of the present study provide varying degrees of evidence for endocrine-mediated effects in fish that were due to possible interactions of toxic and endocrine impacts (Fig. 1). Nonetheless, the proposed combination of in vitro and in vivo tests overall strongly supports the plausibility of endocrine disruption in the test river, which results from chemicals that were not detected or detected only in low concentrations by our chemical analyses.
